# Aberrant brain functional network in COPD patients with cognitive impairment: clinical manifestations, mechanisms and therapeutic strategies

**DOI:** 10.1186/s12974-025-03651-9

**Published:** 2025-12-20

**Authors:** Jia-kai He, Xin-yu Han, Yun-sheng Tan, Zi-ang Yao, Yuan-li Dong, Cui-ling Feng

**Affiliations:** 1 Department of Traditional Chinese Medicine, Peking University People’s Hospital, Beijing, China; 2 Qi-Huang Chinese Medicine School, Beijing University of Chinese Medicine, Beijing, China

**Keywords:** Chronic obstructive pulmonary disease (COPD), Cognitive impairment (CI), Functional magnetic resonance imaging (fMRI), Functional brain network, Neuroinflammation, Lung-brain axis

## Abstract

Cognitive impairment is a prevalent extrapulmonary manifestation of COPD. However, existing reviews have not yet systematically linked COPD-related dysregulation of brain functional networks with clinical indicators. This review bridges this gap by elucidating the pathway from pulmonary pathology to cognitive deficits via central network dysfunction, synthesizing evidence across four dimensions: clinical manifestations; physiological and pathological mechanisms; fMRI-based brain network disorders; and promising treatments. Chronic hypoxia-induced neuroinflammation, oxidative stress, and systemic inflammation propagated via the lung-brain axis were the main pathogenesis of COPD-CI. Cognitive deficits in COPD patients primarily manifest as executive function and visuospatial impairment, with some reality distinctive neural network features showing aberrant functional connectivity between the default mode network and visual network. Long-term oxygen therapy, anti-inflammatory regimens, and cognitive rehabilitation demonstrate benefits in improving cognition. Large sample, cross-sectional study is needed in the future studies, and multimodal neuroimaging should be used to delineate spatiotemporal network dynamics in COPD-CI.

## Introduction

Cognitive impairment (CI) is one of the most prevalent extrapulmonary manifestations of chronic obstructive pulmonary disease (COPD) [1–3], and its prevalence and severity vary depending on the assessment method. The risk increases in patients with hypoxemia [4], severe cases [5, 6], and those diagnosed in middle age [7]. This condition primarily involves deficits in attention, executive function, and other cognitive domains [8, 9]. CI significantly reduces patients’ quality of life, treatment adherence, and clinical prognosis, although its neural mechanisms remain unclear. Neuroimaging techniques such as functional magnetic resonance imaging (fMRI) have preliminarily characterized dynamic neural activity patterns during task executive function and information processing [10]. COPD-related CI (COPD-CI) is associated primarily with aberrant functional coordination within and between brain networks, particularly the default mode network (DMN) [11–13] and the visual network (VN) [11, 12, 14]. Through the aspect of functional connectivity (FC) studies in COPD-CI, this work synthesizes current neuroimaging evidence underlying this comorbidity. In this review, we searched international databases including PubMed, Google Scholar, and Web of Science from June 1992 (the time of publication of the first article on fMRI literature [15]) to July 2025, using search terms “COPD”, “fMRI”, “inflammation” and “cognitive dysfunction” to synthesize functional neuroimaging evidence in COPD-CI. We delineated cognitive deficit profiles in COPD-CI patients, outlined key pathways affecting cognition, and highlighted intra- and internetwork abnormalities in brain FC. Potential therapeutic strategies to mitigate cognitive decline have been proposed. Importantly, we innovatively integrate dispersed multimodal neuroimaging findings into a coherent analysis of key networks like the DMN and VN, transcending the limitations of isolated reports. By emphasizing dysfunctional internetwork interactions as the core mechanism, we elevate the perspective from regional abnormalities to a system-level understanding of whole-brain network synergy, thereby providing a novel theoretical model for COPD-CI and informing future intervention targets.

## Cognitive impairment in COPD: Domain-Specific deficits

### Executive function and decision-making

COPD-CI exhibits domain-specific cognitive deficits. Research consistently indicates that COPD-CI primarily involves impairments in planning, decision-making, attentional allocation, and cognitive flexibility [16]. Compromised visuospatial executive function, attention, abstraction, delayed recall, and orientation were found in COPD patients [9]. A non-amnestic pattern of cognitive dysfunction during stable COPD phases is predominantly characterized by executive dysfunction. These frontal lobe-predominant cognitive profiles may stem from structural damage in prefrontal regions among COPD patients [17]. Different from Alzheimer’s disease (AD), early-stage episodic memory decline is characterized, which is associated primarily with medial temporal lobe pathology involving the entorhinal cortex and hippocampus in patients with AD [18].

### Visuospatial impairment

Visuospatial deficits represent a prominent clinical feature in patients with COPD-CI. A study employing executive visual reproduction tasks demonstrated compromised task completion efficiency and marked impairment in figure retention among COPD-CI patients [19]. Another study revealed lower scores of Montreal Cognitive Assessment (MoCA) in visuospatial construction, sustained attention, and task-switching functions in COPD-CI patients than in healthy individuals [16].

### COPD severity-cognition correlation

The severity of CI is correlated with the severity of COPD. Severe-stage COPD patients were reported to have poorer performance on language, visuospatial, and frontal-executive function tests than moderate-stage COPD patients [6]. Employing the same classification, differences were found between the COPD groups (mild, moderate, severe) and the healthy control group in the total MoCA score and its subdomains, including visuospatial/executive, attention, abstraction, delayed recall, and orientation [5]. Similarly, worse performance in verbal abstraction, attention, recall, and orientation was also found in COPD patients who were suffering from acute exacerbations than those who were in stable phases [20]. These findings collectively indicate that progressive cognitive decline parallels COPD progression. Although distinct neuropsychological profiles exist in COPD-CI and AD, some shared mechanisms may underlie their comorbidity [21]. Notably, a study reported that patients with COPD had an adjusted hazard ratio of 1.74 for developing dementia (either AD or PD) compared with control individuals [22].

## Principal pathways linking COPD to cognitive dysfunction

Multifactorial mechanisms lead to COPD-CI. Chronic hypoxia and systemic inflammation were the main pathogenic factors. Chronic hypoxia leads to mitochondrial dysfunction, exacerbates oxidative stress [23] and dysregulates neuronal energy metabolism, and results in disrupted cerebral structural integrity and FC patterns [24]. Systemic inflammation is a hallmark of COPD, which facilitates peripheral cytokine infiltration into the central nervous system (CNS) through direct and indirect routes. Furthermore, dysregulation of the lung-brain axis, recurrent infections, and medication side effects can exacerbate neuroinflammation and metabolic imbalance [25]. These pathological cascades predominantly damage the hippocampus and prefrontal cortex, which are critical regions for cognition, and progressively disrupt FC across associated brain networks, ultimately leading to impaired global cognitive performance. The details are elaborated in the following section.

### Chronic hypoxia-induced neuroinflammation and oxidative stress

Chronic intermittent hypoxia (CIH) triggers oxidative stress and neuroinflammation in brain regions vulnerable to early neurodegeneration [26], thereby accelerating neurodegenerative processes. CIH-driven hippocampal neuronal pyroptosis, synaptic plasticity impairment, and blood-brain barrier (BBB) disruption constitute key mechanisms underlying these deficits [27, 28]. CIH is a common pathological feature in respiratory disorders. Nocturnal CIH severity independently predicts dementia risk in COPD patients [29]. Positive correlations between arterial oxygen partial pressure (PaO₂) and visuospatial task performance were found in COPD patients [19], suggesting that hypoxia is a critical contributor to cognitive impairment. In support of this, a study found that cognitive deficit severity was negatively correlated with PaO₂ levels in COPD patients [9]. Further, in a neuroimaging study, the amplitude of low-frequency fluctuation (ALFF) was employed to reflect spontaneous neural activity during the resting state. The findings demonstrated that reduced ALFF values in the bilateral basal ganglia and right thalamus were positively correlated with PaO₂ levels in COPD patients [30].

### Systemic inflammation associated with COPD invasion of the CNS

#### How does peripheral inflammation invade the CNS

COPD triggers chronic airway inflammation and pulmonary parenchymal destruction [31]. Inflammatory cytokines serve as critical contributors to CI in COPD patients. Immune cells can activate multiple pathways, including the NOD-like receptor protein 3 inflammasome and nuclear factor kappa-light-chain-enhancer of activated B cells (NF-κB) signalling, and subsequently release downstream inflammatory mediators such as Interleukin-1β (IL-1β), tumour necrosis factor-α (TNF-α), Interleukin-8 (IL-8), and Interleukin-18 [32]. These cytokines drive pathological processes, including airway epithelial hyperplasia, excessive mucus secretion, and epithelial-mesenchymal transition [33]. There are elevated serum levels of C-reactive protein, fibrinogen, serum amyloid A, and proinflammatory cytokines in COPD patients [34, 35]. This biochemical profile indicates the progression from localized pulmonary inflammation to a generalized systemic inflammation. Systemic inflammation distributed throughout the body and can cross the BBB through three main pathways: direct, indirect, and vagal nerve-mediated mechanisms. Specifically, as follows:

##### Direct pathways: systemic inflammation exacerbates neurological impacts

Systemic inflammation not only accelerates COPD progression but also facilitates the systemic dissemination of inflammatory mediators through the circulatory system, compromising the integrity and affecting the permeability of the BBB [36]. Two distinct pathways for the entry of specific inflammatory cytokines (TNF-α and IL-1β) into brain tissue have been identified. These mediators can traverse the BBB either through direct penetration or via selective transport mechanisms. The latter process involves interactions with specific receptors (e.g., Tumour necrosis factor receptor 1, Tumour necrosis factor receptor 2 and Interleukin-1 receptor 1) and caveolin-mediated endocytosis pathways located in cerebral endothelial cells [37]. In the CNS, these cytokines activate the NF-κB pathway and modulate neuronal apoptosis [38] and disrupt the structural and functional homeostasis of neuronal and glial cells [38, 39]. This dual mechanism perpetuates the inflammatory cascade, establishing a self-reinforcing cycle of neuroinflammation.

##### Indirect pathways: neuroglia-mediated inflammatory cascades

Systemic inflammatory mediators breach the BBB to activate microglia, triggering their transformation into a proinflammatory phenotype. Glial cells, a major nonneuronal cell population in the CNS, play a pivotal regulatory role in cerebral inflammatory responses. Activated microglia release cytokines, reactive oxygen species (ROS), and adhesion molecules [40], which exacerbate axonal/synaptic damage, promote demyelination, and impair white matter integrity across multiple brain regions [41]. Glia-derived cytokines, chemokines, and ROS further disrupt BBB integrity while reactivating neighbouring glial cells, establishing a self-perpetuating inflammatory cycle that amplifies neurological deterioration [42].

##### Autonomic effects: vagus nerve-mediated inflammatory pathways

Peripheral cytokines directly stimulate vagal afferent fibres, establishing a conduit for peripheral immune signals to the CNS [43]. The lungs are densely innervated by vagal terminals expressing diverse sensory receptors, including transient receptor potential vanilloid 1 and purinergic P2X receptors [44, 45]. In COPD patients, lung-resident immune cells release inflammatory cytokines, including TNF-α, IL-1β, and Interleukin-6 (IL-6), which activate their receptors and initiate action potentials [46]. These signals propagate as electrical impulses along vagal afferents to brainstem nuclei, projecting to the nucleus tractus solitarius and synapsing with secondary neurons for subsequent transmission to broader cortical regions [47]. Current neuroanatomical studies have demonstrated that transcutaneous vagus nerve stimulation significantly modulates the emotional hubs, including the medial prefrontal cortex (mPFC), dorsolateral prefrontal cortex (dlPFC), anterior cingulate cortex (ACC), insula, and precuneus [48], which were pathophysiologically implicated in COPD-CI.

In summary, systemic inflammation triggered by COPD transmits peripheral inflammatory signals to the CNS through three principal pathways, namely, direct cytokine infiltration, indirect neuroglial activation, and vagus nerve-mediated pathway. This neuroinflammatory milieu induces central inflammation, driving synaptic neurotransmitter dysregulation, disrupting neural metabolic homeostasis, and provoking functional disorganization within and between critical brain regions. These cumulative pathophysiological alterations ultimately manifest as clinically significant CI.

#### Brain regions involved in the systemic inflammatory response contribute to CI

Widespread neuroinflammation across the whole brain was found in animal models of COPD in early years. After cigarette smoke exposure (CSE), levels of interferon-γ, TNF-α, and IL-1β and the corresponding mRNA levels elevated in brain lysates [49]. However, studies revealed that some specific brain regions were particularly susceptible to neuroinflammation and associated with CI [50]. The cerebral cortex is the core region for processing cognition such as perception, motor control, language processing, memory storage, decision-making, and attention regulation. Pro-inflammatory cytokine TNF-α was highly expressed in the cortical slices of CSE mice [51].

The hippocampus primarily facilitates the conversion of short-term memory to long-term memory, particularly supporting the formation of episodic and spatial memories. CSE leads to significant upregulation of pro-inflammatory factors (TNF-α, IL-1β), chemokines, and matrix metalloproteinases in the hippocampus [52]. Similarly, 24 weeks of CSE reduced the number of microglia and the total length of their processes in the CA3 region of the hippocampus, accompanied by poor performance in novel object recognition and Y-maze tests [53]. Further, CSE can induce reactive astrocyte phenotypic transformation in both the hippocampus and hypothalamus [53, 54].

The amygdala is a hub for emotional processing. Through its interaction with the hippocampus, the amygdala enhances the encoding and storage of emotionally salient memories [55]. Furthermore, it participates in decision-making, risk assessment, and social behavioural judgments, working in concert with the prefrontal cortex to modulate complex cognitive processes [56]. CSE upregulated the mRNA expression of the pro-inflammatory markers IL-6, IL-1β, and TNF-α only in the amygdala. Concurrently, it reduced the number of microglia marked by ionized calcium-binding adapter molecule 1 positivity in this region, while no similar changes were observed in the other brain areas examined such as the hippocampus and prefrontal cortex [57].

In summary, the cerebral cortex, hippocampus and amygdala are more susceptible to inflammation. Further elucidation of the specific brain regions and mechanisms by which inflammation contributes to cognitive impairment in COPD-CI requires extensive clinical validation.

### Other risk factors associated with CI in COPD patients

#### Smoking

Cigarette smoking has a dose-dependent association with severity of COPD. Prolonged carbon monoxide exposure exacerbates cerebral hypoxia by shifting the oxyhemoglobin dissociation curve and accelerating cognitive decline [58]. An animal study has demonstrated that tobacco-exposed mice exhibit significant cognitive deficits in behavioural tests such as the Y-maze and brief smoking cessation fails to restore cognitive function in these models [52]. However, recent studies present paradoxical findings regarding the role of smoking in CI severity. It was reported that COPD patients with a smoking history had higher MoCA scores than in non-smokers, and the reduced regional homogeneity (ReHo) values in the left fusiform gyrus are attributed to the neuroprotective effects of nicotine on dopaminergic neurons [59]. It was found that nicotine-free e-cigarettes exposure impaired memory retention in rodents during novel object recognition tasks, with detrimental effects exceeding those of conventional nicotine-containing cigarettes [60].

In summary, smoking worsens COPD and cerebral hypoxia, and its impact on cognitive impairment is complex and paradoxical. Some studies suggest nicotine may offer neuroprotection and associate smoking history with better cognitive scores in COPD patients, while others show nicotine-free e-cigarettes cause worse memory impairment than nicotine-containing ones. Further studies should clarify the specific effects of nicotine and other tobacco components on brain structure and function. First, in clinical research, large-scale, multicenter longitudinal studies should be conducted to evaluate the impact of long-term smoking, smoking cessation, and the use of nicotine-containing or nicotine-free alternatives on cognitive function in patients with COPD. These studies should employ more precise cognitive assessment tools, combined with neuropsychological testing and neuroimaging techniques, to elucidate how different types of tobacco exposure influence cognitive function by altering brain network characteristics. Second, in basic research, animal models are essential for mechanistic exploration. Experimental designs could include mouse models exposed to cigarette smoke, e-cigarette vapor, or nicotine alone to compare CI across groups. Particular attention should be paid to their effects on blood-brain barrier integrity, neuroinflammatory responses, and neuronal plasticity. Additionally, genetic heterogeneity among individuals must be considered to identify potential susceptibility genes or protective factors, which could inform personalized prevention and treatment strategies. Such integrated approaches will deepen our understanding of the complex interplay between tobacco exposure and COPD-CI, ultimately guiding the development of targeted interventions.

#### COPD comorbidities exacerbating cognitive dysfunction

COPD is a complex, systemic syndrome that often coexists with obstructive sleep apnoea syndrome (OSAS), cardiovascular diseases, diabetes, and depressive/anxiety disorders [61]. It exerts a synergistic damaging effect on the body’s cognitive function through a complex network mechanism. In addition to hypoxia and inflammatory mechanisms, other mechanism pathways are also being explored and discovered. Cohort studies revealed significantly lower Mini-Mental State Examination (MMSE) scores in COPD patients with comorbid OSAS than in their COPD-only counterparts [29], suggesting that respiratory comorbidities directly impair cognition via hypoxemia-related pathways. Furthermore, metabolic comorbidities such as hypertension and diabetes aggravate cognitive deficits by promoting arteriosclerosis [62], insulin resistance [63] and cerebrovascular microenvironmental disturbances [64, 65]. However, there is still a lack of clinical research on the impact of metabolic complications on cognitive impairment in COPD-CI patients. Cardiovascular comorbidities reduce cerebral perfusion and contribute to cognitive decline [66]. Notably, the prevalence of neuropsychiatric comorbidities is particularly high in COPD patients, with depression/anxiety affecting up to 65% of patients [67]. Depression and anxiety can not only predict cognitive impairment but also increase risk of visuospatial and executive dysfunction [68, 69].

**Fig. 1:**
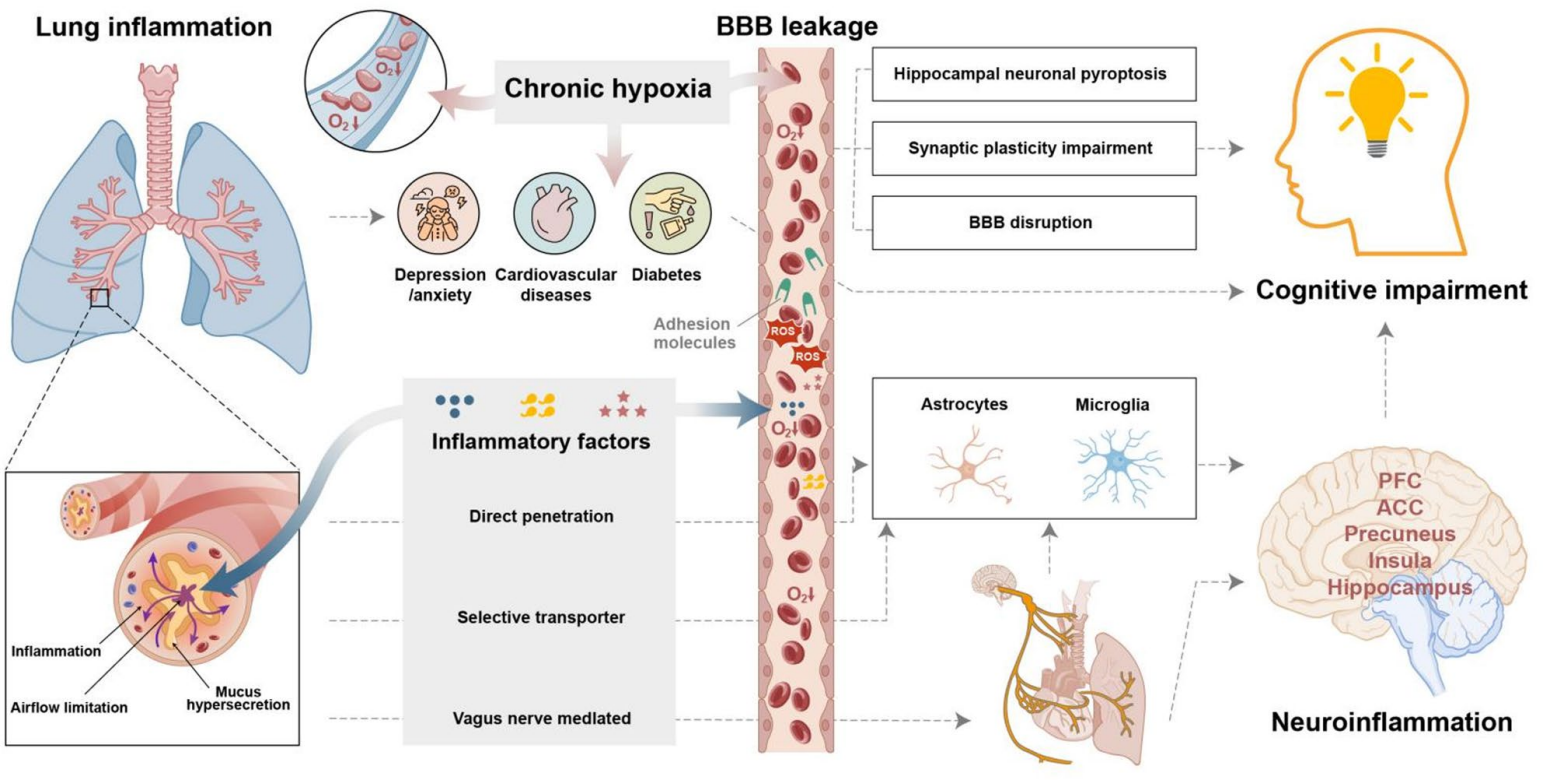
The pathogenic cascade linking COPD to CI. The typical features of COPD are lung inflammation, airflow limitation and hypersecretion of mucus, which leads to chronic intermittent hypoxia and the synergistic effect of inflammatory factors. Chronic hypoxia-induced inflammation and oxidative stress lead to disrupted BBB. COPD raises the risk of depression/anxiety, cardiovascular diseases and diabetes, which are independent risks of CI. The inflammatory factors induce the generation of ROS and adhesion molecules, and they mediate the destruction of the BBB. The inflammatory factors can penetrate the BBB directly, the selective transporters on BBB help to transfer the peripheral inflammatory messages to neuroimmune cells, and the inflammatory factors can influence the vagus nerve afferent pathway, directly amplifying the neuroinflammatory response. Abbreviations: BBB: blood-brain barrier, ROS: reactive oxygen species, ACC: anterior cingulate cortex, PFC: prefrontal cortex, CI: cognitive impairment

In summary, these findings demonstrate that COPD-associated comorbidities exacerbate cognitive decline through multidimensional interactive mechanisms, underscoring the imperative for comprehensive comorbidity management in cognitive protection strategies for COPD patients (Fig. 1).

## Aberrant brain networks in COPD-CI

### Brain functional network and cognition

The brain functional network represents a complex system of interconnected neural regions, where intra- and internetwork interactions underlie the executive function of specific cognitive and behavioural tasks. Key networks governing higher cognition include the DMN, VN, executive control network (ECN), and salience network (SN). The DMN, anchored by nodes such as the medial prefrontal cortex and posterior cingulate cortex, mediates self-referential processing, autobiographic memory, and mental scene construction [70]. The VN specializes in processing and integrating external visual stimuli into cognitive operations. These networks achieve information synthesis through dynamic collaboration: during visual scene analysis, the VN initially processes sensory inputs via intra- network connections and then engages the anterior temporal nodes of the DMN to contextualize visual content with self-referential knowledge [71]. Concurrently, the ECN allocates attentional resources for task-oriented analysis, synergizing with the VN to enable complex scene interpretation and cognitive decision-making [72]. This cross-network integration of internal and external information forms the neurobiological basis of advanced cognitive functions.

Emerging brain network analyses have revealed characteristic network disruptions in neurocognitive disorders. For instance, schizophrenia patients exhibit aberrant DMN-SN connectivity, which is correlated with impaired self-monitoring [73]. In AD, early-stage amyloid deposition and FC attenuation within DMN hubs precede memory-related cognitive deficits [74, 75]. Collectively, these findings underscore that the structural and functional integrity of brain regions/networks is indispensable for maintaining higher-order cognition.

### Aberrant brain functional network in COPD-CI

Impairments within brain functional networks manifest as diverse cognitive deficits. These alterations are closely associated with deficits in attention, executive function, and visuospatial abilities, reflecting the neuropathological cascade linking COPD-specific mechanisms to cognitive decline.The DMN is mainly composed of the medial prefrontal cortex, posterior cingulate cortex (PCC), and precuneus [76], which integrates memory, language, and semantic representations [70]. The VN mainly encompasses the occipital cortex and inferior temporal gyrus, which process visual information and object recognition. Tables 1 and 2 summarize studies of altered networks in COPD-CI and clinical correlation.

**Table 1:** Studies focusing on altered inter or intra network in COPD-CI with summarized main findings and clinical correlation

Specific Brain Regions or Functional networks		Functional Metric	Direction	Key Regional Changes	Clinical/Cognitive Correlation	Sample Size	Reference	Associated Cognitive Functions
ROIs of DMN	L-hippocampus	rs-FC	↓	Decreased FC between L-hippocampus and L-posterior cingulate cortex	The FC values between L-hippocampus and L-posterior cingulate cortex were positively correlated with MoCA total score, indicating that patients who have lower FC values between L-hippocampus and L-posterior cingulate cortex performed poorer scores in MoCA total test and visual reproductive test.	113	[9]	Attention, episodic memory, self-referential processing, orientation, visuospatial/executive function, semantic memory, visual memory
	R-medial orbitofrontal cortex	rs-FC	↓	Decreased FC between R-medial orbitofrontal cortex and L-posterior cingulate cortex	The FC values between R-medial orbitofrontal cortex and L-posterior cingulate cortex were positively correlated with orientation scores, indicating that patients who have lower FC values between R-medial orbitofrontal cortex and L-posterior cingulate cortex performed poorer scores in orientation test.			
	R-parahippocampus	rs-FC	↓	Decreased FC between R-parahippocampus and L-posterior cingulate cortex	The FC values between R-parahippocampus and L-posterior cingulate cortex were negatively correlated with the naming function score, indicating that patients who have lower FC values between R-parahippocampus and L-posterior cingulate cortex performed poorer scores in naming function test.			
	L-posterior cingulate cortex	FC	↓	Decreased FC between L-posterior cingulate cortex and multiple regions (L-posterior cingulate cortex, L-anterior cingulate cortex, L-supramarginal gyrus, L-hippocampus, R-parahippocampus, R-medial orbitofrontal cortex)	The FC values between L-posterior cingulate cortex and multiple regions were positively correlated with visuospatial/executive function, indicating that patients who have lower FC values between L-posterior cingulate cortex and multiple regions performed poorer scores in visuospatial/executive test. The FC values of L-posterior cingulate cortex, L-anterior cingulate cortex, L-hippocampus and R-medial orbitofrontal cortex were positively correlated with the FEV1 of patients, indicating that patients who have lower FC values of L-posterior cingulate cortex, L-anterior cingulate cortex, L-hippocampus and R-medial orbitofrontal cortex had poorer lung function.			
ROIs of VN	Lingual gyrus	FC	↓	Decreased FC between L-lingual gyrus and R-lingual gyrus	/	39	[14]	Visual processing, visual reproduction, figure retention, visual-verbal integration.
	L-cuneus gyrus	FC	↑	Increased FC between R-middle occipital gyrus and L-cuneus gyrus	/	85	[77]	
	L-fusiform gyrus	FC	↓	Decreased FC between L-fusiform gyrus and R-middle occipital gyrus	/			
	R-middle occipital gyrus	FC	↓	Decreased FC between R-middle occipital gyrus and L-fusiform gyrus	The FC values between R-middle occipital gyrus and L-fusiform gyrus were positively correlated with MoCA, language-domain score, indicating that patients who have lower FC values between R-middle occipital gyrus and L-fusiform gyrus performed poorer in MoCA and language-domain test.			
ROIs of Other Networks	R-supramarginal gyrus and L-dorsolateral prefrontal cortex/insula	FC	↓	Decreased FC between R-supramarginal gyrus and L-dorsolateral prefrontal cortex/insula	The FC values between R-supramarginal gyrus and L-dorsolateral prefrontal cortex/insula were positively correlated with attention, indicating that patients who have lower FC values between R-supramarginal gyrus and L-dorsolateral prefrontal cortex/insula performed poorer in attention test.			Dysfunctional synergy across the VN, frontoparietal, and DMN networks
	L-supramarginal gyrus	FC	↓	Decreased FC between L-supramarginal gyrus and L-posterior cingulate cortex; Decreased FC between L-supramarginal gyrus/precentral gyrus and L-dorsolateral prefrontal cortex	1.The FC values between L-supramarginal gyrus and L-posterior cingulate cortex were negatively correlated with visuospatial/executive function, indicating that patients who have higher FC values between L-supramarginal gyrus and L-posterior cingulate cortex performed poorer in visuospatial/executive function test. 2.The FC values between L-supramarginal gyrus/precentral gyrus and L-dorsolateral prefrontal cortex were positively correlated with COPD disease duration, indicating that patients who have higher FC values between L-supramarginal gyrus/precentral gyrus and L-dorsolateral prefrontal cortex have experienced a longer disease course.	113; 85	[9]; [77]	
FNC	ECN-DMN	Dynamic FNC	-	Across multiple states, COPD patients consistently exhibit enhanced connectivity between the ECN, DMN, and VN as the predominant dysfunction.	Fractional windows, mean dwell time in States II and III (the strong connectivity) were positively associated with FEV1 and FEV1/FVC, indicating that longer time spent in these states was related with lower lung COPD severity. The dFNC temporal characteristics in State I (the weak connectivity and the long mean dwell time) were negatively correlated with FEV1, FEV1/FVC and pH and positively correlated with CRP.	38	[78]	Overall, these abnormal functional connections mainly occurred in DMN, ECN, and VN.
	DMN, ECN, LN, SMN, VN, SN, AN	rs-FNC	↓	Reduced coupling between the DMN-VN, ECN-SMN, VN-LN, and VN-SMN,	/			
			↑	Increased coupling between the ECN-SN, ECN-VN, DMN-LN, DMN-SMN, DMN-ECN, and AN-VN	/			

Table 1 comprehensively summarizes key alterations in brain functional network connectivity in patients with COPD-CI. Studies have revealed extensive abnormalities in functional connectivity, both within core networks such as the DMN and VN, and between multiple networks including the DMN, ECN, and VN. These alterations are primarily characterized by weakened intra-network connectivity and increased/decreased inter-network connectivity, which are significantly correlated with declines in patients’ cognitive function scores and worsening lung function. Collectively, these changes represent a key neuropathological basis of COPD-CI

*Abbreviations*: *DMN* default mode network, *VN* visual network, *SN* salience network, *ECN* executive control network, *LN* language network, *SMN* sensorimotor network, *AN* auditory network, *ROI* region of interest, *FC* functional connectivity, *rs-FC* resting-state functional connectivity, *FNC* functional network connectivity, *rs-FNC* resting-state functional network connectivity, *dFNC* dynamic functional network connectivity, *L* left, *R* right, *PCC* posterior cingulate cortex, *ACC* anterior cingulate cortex, *mOFC* medial orbitofrontal cortex, *PHC* parahippocampal cortex, *SMG* supramarginal gyrus, *MOG *middle occipital gyrus, *DLPFC* dorsolateral prefrontal cortex, *MoCA* Montreal Cognitive Assessment, *FEV1* forced expiratory volume in 1 s, *FVC* forced vital capacity, *CRP* C-reactive protein

**Table 2:** Studies focusing on altered local brain regions in COPD-CI with summarized main findings and clinical correlation

Brain Network	Specific Brain Region	Functional Metric	Direction	Key Regional Changes compared with HCs	Clinical Correlation	Sample Size	Reference
DMN	Bilateral posterior cingulate cortex	ALFF	↓	Decreased ALFF in bilateral posterior cingulate cortex	1. The ALFF values in bilateral posterior cingulate cortex were positively correlated with the patients’ visual reproductive scores, indicating that patients who have lower ALFF values in the bilateral posterior cingulate cortex performed poorer scores in visual reproductive test. 2. The ALFF values in bilateral posterior cingulate cortex were positively correlated with the patients’ FEV1/FVC values, and negatively correlated with the patients’ PaCO_2_ values, indicating that patients who have lower ALFF values in bilateral posterior cingulate cortex had poorer lung function and higher levels of carbon dioxide retention.	50; 39	[12]; [13]
	Bilateral precuneus	ReHo/ALFF	↓	Decreased ReHo and ALFF in bilateral precuneus	1. The ALFF values in bilateral precuneus were negatively correlated with the patients’ PaCO_2_ values, indicating that patients who have lower ALFF values in bilateral precuneus had higher levels of carbon dioxide retention. 2. The ReHo values in bilateral precuneus were positively correlated with the patients’ FEV1% and FEV1/FVC levels, as well as the patients’ orientational function, indicating that patients who have lower ReHo values in bilateral precuneus had poorer lung function and performed poorer in orientational function. 3. The ReHo values in bilateral precuneus were negatively correlated with the patients’ PaCO_2_ values, indicating that patients who have lower ReHo values in bilateral precuneus had higher levels of carbon dioxide retention.	39; 39	[13]; []
	L-supramarginal gyrus	DC	↑	Increased DC in L-supramarginal gyrus	The DC values in L-supramarginal gyrus were negatively correlated with the patients’ MoCA total score and visuospatial/executive scores, indicating that patients who have lower DC values in L-supramarginal gyrus performed poorer scores in MoCA total test and visuospatial/executive test.	85	[79]
	Bilateral hippocampal/parahippocampal cortex	dALFF	↓	Decreased dALFF in bilateral hippocampal/parahippocampal cortex	1. The dALFF values in the L-hippocampal/parahippocampal cortex were negatively correlated with semantic-memory performance, indicating that patients who have higher dALFF values in the L-hippocampal/parahippocampal cortex performed poorer in category verbal fluency test. 2. The dALFF values in the R-hippocampal/parahippocampal cortex were negatively correlated with the patients’ FVC level, indicating that patients who have higher dALFF values in the R-hippocampal/parahippocampal cortex had poorer lung function.	62	[80]
VN	L-occipital cortex	ReHo	↓	Decreased ReHo in L-occipital cortex	/	39	[11]
	R-lingual gyrus	ALFF/ReHo/DC	↓	Decreased ALFF, ReHo, and DC in R-lingual gyrus	The ALFF values in R-lingual gyrus were positively correlated with the patients’ visual reproduction score, indicating that patients who have lower ALFF values in R-lingual gyrus performed poorer in visual reproductive test.	39; 50; 39	[12]; [11]; [14]
Other Networks	Supplementary motor area	DC	↓	Decreased DC in supplementary motor area	The DC values in supplementary motor area were positively correlated with the patients’ naming score and pH level of arterial blood, indicating that patients who have lower DC values in supplementary motor area exhibited poorer naming performance and a greater tendency toward acidosis.	39	[14]
	R-paracentral lobule	DC	↓	Decreased DC in R-paracentral lobule	The DC values in R-paracentral lobule were positively correlated with the patients’ FEV1% and FEV1/FVC levels, indicating that patients who have lower DC values in R-paracentral lobule had poorer lung function.		
	L-basal ganglia; bilateral basal ganglia	ALFF	↓	Decreased ALFF in basal ganglia	The ALFF values in L-basal ganglia were positively correlated with the patients’ PaO_2_ values, indicating that patients who have lower ALFF values in L-basal ganglia had poorer systemic oxygenation.	62; 54	[80]; [30]
	R-thalamus	ALFF	↓	Decreased ALFF in R-thalamus	/	54	[30]
	L-postcentral gyrus	ALFF	↑	Increased ALFF in L-postcentral gyrus	/	50	[12]
	Brainstem	ALFF	↑	Increased ALFF in brainstem	The ALFF values in brainstem were positively correlated with the patients’ FEV1 level, indicating that patients who have lower ALFF values in brainstem had poorer lung function.	39	[13]
	L-precentral gyrus	DC	↑	Increased DC in L-precentral gyrus	/	85	[79]

Table 2 shows in patients with COPD-CI, the entire cerebral cortex presents a hypometabolic state, and the activity of most relevant brain regions is significantly reduced. Generally, the activity of these brain regions exhibits a positive correlation with blood oxygen levels and a negative correlation with the PaCO₂ level, leading to a state of cerebral hypoxia and hypercapnia. Furthermore, the activity of these brain regions also shows a positive correlation with cognitive function-related scores. Based on this, it can be inferred that the impairment of pulmonary ventilation function in COPD patients maintains a prolonged state of hypoxia and hypercapnia in the body. This pathological state can contribute to the decrease in the activity of relevant brain regions, ultimately leading to cognitive decline

*Abbreviations*: *DMN* default mode network, *VN* visual network, *ALFF* amplitude of low-frequency fluctuation, *ReHo* regional homogeneity, *DC* degree centrality, *dALFF* dynamic amplitude of low-frequency fluctuation, *FEV1* forced expiratory volume in 1 second, *FVC* forced vital capacity, *PaCO*_*2*_ arterial partial pressure of carbon dioxide, *MoCA* Montreal Cognitive Assessment, *PaO*_*2*_ arterial partial pressure of oxygen, *L* left, *R* right, *HCs* healthy controls

#### Abnormalities in DMN

Reduced activation volumes within DMN were found in the COPD. In the study of Hu et al. four cohorts of participants were stratified: healthy controls and mild, moderate, and severe COPD patients. It was found that the COPD group showed reduced activation volumes within the DMN compared with the control group, with the most pronounced reductions observed in the severe COPD group [9]. Decreased ReHo values were found in the bilateral precuneus of COPD patients compared with healthy controls [11]. Reduced ALFF values were reported in the bilateral PCC [12], whereas another study reported elevated ALFF in the brainstem alongside diminished ALFF in the PCC and precuneus [13]. Notably, the PCC and precuneus were core DMN hubs, orchestrating episodic memory retrieval, information evaluation, mind wandering, and attentional processing [81]. Consequently, PCC/precuneus dysregulation may underlie the executive and decision-making impairments in COPD-related cognitive dysfunction, specifically through disrupted information evaluation and attentional control.

Neural activity and interregional functional coupling within the DMN in COPD patients were associated with cognitive impairment, highlighting network-specific pathophysiological signatures [13]. Using the left posterior cingulate cortex (L-PCC) as a seed, they identified significant differences in FC strength between the L-PCC and six key DMN nodes, namely, the L-PCC, left anterior cingulate cortex, left supramarginal gyrus, left hippocampus, right parahippocampus (R-PHP), and right medial orbitofrontal cortex. MoCA scores were positively correlated with FC values between L-PCC and left hippocampus, whereas their scores on executive functions and visuospatial function were positively correlated with intra-L-PCC FC. Conversely, the patients’ scores on naming ability were inversely correlated with the FC values between R-PHP and L-PCC, and the orientation scores were significantly associated with the FC values between L-PCC and right medial orbitofrontal cortex. In addition to FC metrics, studies employing ReHo and ALFF analyses further delineate DMN dysfunction in COPD patients.

It is noteworthy that elevated DC in the left precentral gyrus (PreCG) and left supramarginal gyrus (SMG) were found in COPD patients, and the DC values in SMG were negatively correlated with MoCA scores and visuospatial/executive function sub-scores [79]. Another study revealed decreased dynamic ALFF (dALFF) in bilateral hippocampal/parahippocampal cortex in COPD patients [80], with the dALFF values negatively correlated with semantic memory performance on the left and with FVC levels on the right. Contrary to the commonly observed pattern of “low ALFF being associated with functional decline,” this inverse relationship may indicate a compensatory suppression within the hippocampal/parahippocampal regions, whereby reduced activity helps maintain stability in cognitive and pulmonary function. These parallel findings demonstrate that dysfunctional cross-nodal coupling within the cortico-basal ganglia-thalamic circuit implicates multinetwork dysregulation in COPD-related cognitive pathology.

#### VN abnormalities

The visual network has emerged as a critical locus of neurofunctional aberrations in COPD patients with cognitive impairment. Reduced ReHo, ALFF and degree centrality (DC) values in the lingual gyrus were found in COPD patients [11, 12, 14]. The reduced ALFF values in the right lingual gyrus were positively correlated with visual sub-scores of the Chinese Wechsler Memory Scale [12]. The three studies revealed an aberrant neural activity in the primary visual cortex. Further, reduced FC between the left lingual gyrus and left fusiform gyrus indicated global deterioration of intra-VN information transfer efficiency [14]. Another study found that FC between the right middle occipital gyrus and left fusiform gyrus was positively correlated with total MoCA scores and language domain performance [77]. A study using dynamic functional network connectivity (dFNC) analysis further confirmed a reduced temporal synergistic efficiency between the VN and SN, revealing the pivotal role of VN functional instability in COPD-related cognitive deficits across temporal dimensions [78].

#### Other network abnormalities

COPD patients exhibit FC abnormalities in the sensorimotor network (SMN), frontoparietal networks, and cortical-basal ganglia-thalamic circuits. For example, reduced cortical thickness was reported in the dlPFC of COPD patients, which leads to frontoparietal network-mediated visuospatial deficits [17]. In COPD patients, reduced DC in the supplementary motor area (SMA) and right paracentral lobule [14], and increased ALFF were found in the left postcentral gyrus [12], which indicate aberrations within the SMN. Decreased ALFF was found in the bilateral basal ganglia and right thalamus [30, 80]. In summary, chronic hypoxia and systemic inflammation in COPD patients lead to reduced global FCs across brain networks [82], characterized by weakened intra-network FC in the DMN and VN [78], decreased DC of hub nodes, and abnormal dynamic network mean dwell time [9].

### Internetwork functional dysregulation in COPD-CI

Cross-network dysregulations were found in COPD patients. Reduced FC between the SMA, right paracentral lobule (R-PCL), and many key regions of the DMN, including the cingulate gyrus and precuneus were found in a study of Li et al. The study suggests an impaired cross-network integration among the sensorimotor, DMN, and limbic networks in COPD [14], with positive correlations between attention subdomain scores on the MoCA and the right SMG to left dlPFC/insula FCs [77]. These findings indicate that dysfunctional synergy across the VN, FPN, and DMN networks may directly drive cognitive deficits, highlighting multinetwork discoordination as a neural substrate of COPD-CI. Dynamic functional network connectivity (dFNC) reflects transient and recurring whole-brain temporal coupling modes through short-term fluctuations [83]. Using dFNC clustering, COPD patients spent minimal time in States II/III characterized by strong ECN-DMN FC, a phenomenon designated as strong connectivity escape, likely hindering effective cross-network integration [78]. Further, widespread alterations in functional network connectivity (FNC) were observed, including reduced coupling between the DMN-VN, ECN-SMN, VN-language network (LN), and VN-SMN, alongside increased coupling between the ECN-SN, ECN-VN, DMN-LN, DMN-SMN, DMN-ECN, and auditory network (AN)-VN. These alterations reflect a state of global network dysregulation. The static-dynamic dual-dimensional findings systematically establish a multinetwork dyssynergia pathomechanism in COPD-associated CI [78].

**Fig. 2:**
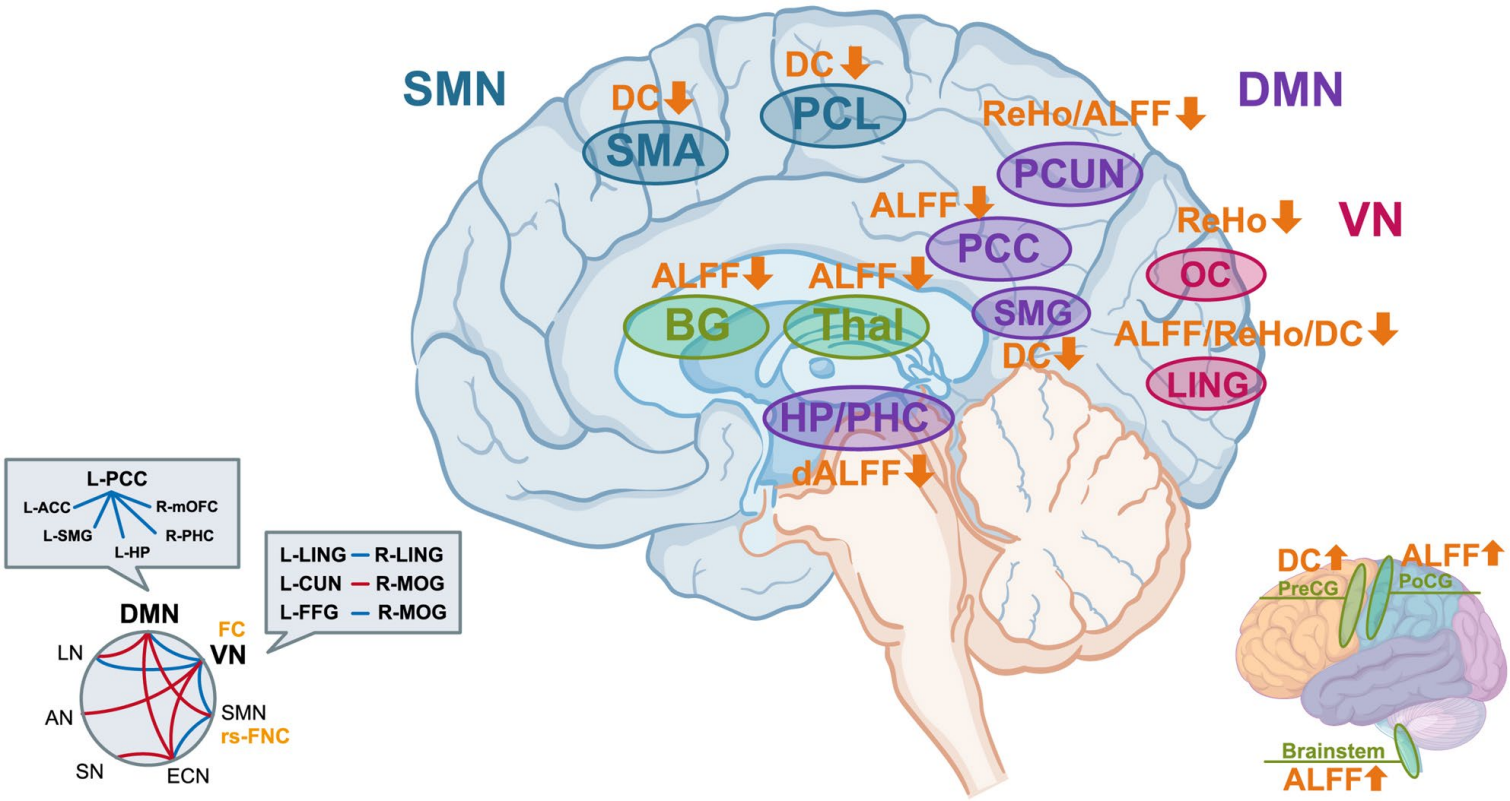
Disrupted functional brain network in COPD-CI. The figure comprehensively illustrates the key brain regions and networks according to the recent studies. Different brain networks are represented by different colors (DMN-purple, VN-red, SMN-blue, LN-green) in the main image. The blue line in the bottom left corner represents a decrease in FC values, while the red line represents an increase in FC values. Widespread reductions of ReHo, ALFF, DC were found in brain regions of DMN, VN, SMN. Aberrant intra- and inter-network connectivity represent a collective impairment that underpins the neuropathological basis of COPD-CI and is likely closely associated with patients' cognitive decline. Abbreviations: DMN: default mode network, VN: visual network, SN: salience network, ECN: executive control network, SMN: sensorimotor network, LN: language network, AN: auditory network, ALFF: amplitude of low-frequency fluctuation, dALFF: dynamic ALFF, ReHo: regional homogeneity, DC: degree centrality, FC: functional connectivity, BG: basal ganglia, Thal: thalamus, PCC: posterior cingulate cortex, ACC: anterior cingulate cortex, PCUN: precuneus, mOFC: medial orbitofrontal cortex, HP: hippocampus, PHC: parahippocampal cortex, SMG: supramarginal gyrus, LING: lingual gyrus, CUN: cuneus, MOG: middle occipital gyrus, FFG: fusiform gyrus, OC: occipital cortex, SMA: supplementary motor area, PCL: paracentral lobule, PreCG: precentral gyrus, PoCG: postcentral gyrus, L: left, R: right

In summary, COPD patients with CI present widespread abnormalities in functional brain networks (Fig. 2). These disruptions show distinct spatial patterns: local functional impairments primarily localize to key hubs of the DMN, particularly the PCC and precuneus, as well as VN regions encompassing the occipital lobe and lingual gyrus. These localized deficits are characterized by synchronized reductions in nodal centrality metrics, including DC, ReHo, and ALFF. Concurrently, interregional FC attenuation coexists with compensatory hyperactivation in specific brain areas, collectively indicating dysregulation of brain network homeostasis. At the cross-network level, aberrant connectivity strength characterizes interactions among the DMN, ECN, and VN, accompanied by a shortened dwell time in low-connectivity states. These multiscale anomalies elucidate the neuropathological underpinnings of COPD-related cognitive deficits, providing critical theoretical foundations for developing targeted clinical interventions.

## Therapeutic potential of selected interventions for COPD-CI

### Hypoxia-targeted therapies

Hypoxia-targeted therapies may mitigate cognitive dysfunction in COPD patients. Long-term oxygen therapy (LTOT), exercise training, and pulmonary rehabilitation were the mainstream hypoxia-targeted therapies. As a fundamental intervention, LTOT has demonstrated efficacy in attenuating cognitive decline across multiple studies. COPD patients receiving LTOT showed a significantly reduced rates of cognitive deterioration compared to non-LTOT group [84]. The underlying neuroprotective mechanisms may involve enhanced cerebral oxygen delivery through sustained improvement in peripheral oxygen saturation via low-flow oxygen therapy and the reversal of chronic hypoxemia [4]. Exercise-based interventions exhibit variable cognitive benefits. An 8-week Tai Chi program combined with standard therapy improved clinical outcomes in COPD patients, which was accompanied by decreased DC values in the right inferior frontal gyrus. The decrease in DC values was positively correlated with symptom improvement, as assessed by lower COPD Assessment Test (CAT) scores [85]. Four-week exercise elevated MoCA scores in COPD patients [86]. Pulmonary rehabilitation is a cornerstone COPD therapy. A study demonstrated that participants exhibited improvements in verbal fluency and visuospatial abilities, along with enhanced global cognitive function relative to baseline measurement [87]. Further, 1-year exercise adherent participants maintained gains they had achieved in the initial exercise intervention, but non-exercise participants experienced declines in functional capacity, cognitive performance, and psychological well-being [88]. In summary, sustained exercise plays a potential role in preventing cognitive deterioration, and its efficacy in enhancing function requires further validation.

### Anti-inflammatory therapeutic approaches

Preclinical studies suggest that select anti-inflammatory agents can alleviate CI in COPD patients through immunomodulatory mechanisms. For example, liraglutide mitigates cognitive deficits in chronic hypoxic murine models by activating the nuclear factor erythroid 2-related factor 2/heme oxygenase-1 axis while suppressing the mitogen-activated protein kinase/NF-κB signaling pathways [89]. Omega-3 polyunsaturated fatty acids reduce the serum levels of high-sensitivity C-reactive protein, IL-6, IL-8, and TNF-α in COPD patients [90]. An animal study suggests that the neuroprotective effects of omega-3 occur via NF-κB inhibition, which downregulates the expression of IL-1β and TNF-α and attenuates neuroinflammation mediated by glial cell activation [91]. Notably, roflumilast demonstrated efficacy in reducing exacerbation risk and improving pulmonary function. A study found that roflumilast can enhance cognition via the cyclic adenosine monophosphate/protein kinase A/cAMP response element-binding protein (cAMP/PKA/CREB) pathway by increasing cAMP levels and activating PKA to phosphorylate CREB, thereby increasing the expression of brain-derived neurotrophic factor (BDNF). This pathway is correlated with reduced neuroinflammation and improved cognitive performance in APP/PS1 transgenic mice [92]. However, there is a lack of relevant research on anti-inflammatory treatment-improvement of functional brain networks-improvement of clinical symptoms.

### Adjunctive therapeutic strategies

In addition to aetiological treatments, pharmacological agents, music therapy, and cognitive training are promising to alleviate the clinical manifestations of COPD-CI. A triple-blind clinical trial demonstrated that Rosmarinus officinalis aqueous alcohol improved MoCA-B scores in COPD patients [93]. Essential amino acid supplementation potentially can improve both quality of life and cognitive performance in patients with severe COPD who are ineligible for conventional rehabilitation programs [94]. Music therapy has emerged as a viable neuromodulation strategy. A meta-analysis revealed that COPD patients receiving music therapy exhibited significant improvements in Pittsburgh Sleep Quality Index Scores, along with reduced depression, anxiety, and dyspnoea severity [95]. Cognitive rehabilitation demonstrated sustained cognitive gains on Mild Vascular Cognitive Impairment Scale immediately postintervention and at the 4-week follow-up [96].

### Clinical implications of network dysfunction

#### Current clinical reality and the potential role of network biomarkers

At present, the diagnosis of COPD-CI primarily relies on neuropsychological scales like the MoCA and MMSE, which are susceptible to confounding factors such as education level and cultural background. Although alterations in the DMN and VN have been identified in COPD patients, their direct application in clinical diagnosis, prognosis, or treatment guidance remains a future goal rather than a current reality. In this context, the specific changes observed in the DMN and VN can serve as crucial objective biomarkers to supplement these clinical assessments. They hold significant potential for aiding the differential diagnosis of COPD-CI by helping to distinguish it from primary neurodegenerative disorders like Alzheimer’s disease, which may exhibit distinct neural network signatures. Furthermore, while their prognostic value is currently limited by a lack of longitudinal studies, we propose that these functional network metrics could evolve into sensitive tools for monitoring treatment efficacy. They may detect subtle, treatment-induced neuroprotective effects that precede measurable changes on conventional cognitive scales, thereby enabling a more precise and objective evaluation of therapeutic interventions in future clinical practice and research.

#### Challenges hindering clinical translation

Although we anticipate that DMN and VN metrics could serve as biomarkers for early intervention, their direct application in clinical practice remains premature, primarily due to challenges across three dimensions. First and foremost, there is a fundamental lack of longitudinal evidence: most existing studies are based on cross-sectional designs, which cannot establish causality with cognitive decline, and large-scale prospective cohort studies are still needed to verify whether these indicators can reliably predict future disease progression. Secondly, on the technical translation front, the absence of standardization poses a significant hurdle—no universally accepted “abnormality threshold” for diagnosing COPD-CI based on brain networks has been established, leading to inconsistent interpretation of results. Finally, considerations of cost and accessibility present practical barriers, as fMRI is expensive and operationally complex, making it difficult to implement as a routine screening tool for the broader COPD population.

#### A novel therapeutic direction: network-targeted interventions

Currently, no interventional studies have directly employed neuromodulation techniques to target specific brain networks for the explicit purpose of enhancing cognitive performance in COPD-CI. However, a compelling scientific rationale for this approach is built upon a growing body of successful precedents across various neurological and psychiatric conditions. The core concept is that non-invasive brain stimulation techniques can selectively modulate pathological network activity to improve cognitive and behavioral outcomes. Non-invasive brain stimulation techniques, such as transcranial magnetic stimulation and transcranial direct current stimulation, can directly modulate dysfunctional neural networks including in patients with major depressive disorder [97, 98], AD [99]and post-stroke CI [100]. Building on this precedent, we propose that selectively targeting aberrant brain networks represents a promising and novel therapeutic direction for COPD-CI. Future research should therefore explore whether modulating the dysfunctional connectivity identified in COPD patients can translate into tangible cognitive benefits.

## Conclusions

This review synthesizes cerebral functional network abnormalities and the underlying mechanisms in COPD patients with CI, revealing that chronic hypoxia and systemic inflammation mediate brain network dysregulation through dysregulated lung-brain axis interactions. Studies have demonstrated that COPD-associated cognitive deficits are characterized by impaired attention, executive function (including planning, decision-making, cognitive flexibility), and visuospatial decline. To elucidate the neural mechanisms underlying these impairments, we propose a framework centred on specific disruption patterns within functional brain networks. Specifically, the DMN exhibits abnormalities in local regional activity and disrupted functional coupling between brain regions. These alterations manifest as: decreased ReHo and ALFF values in the PCC and Precuneus; elevated ALFF in the brainstem; decreased dALFF values in the bilateral hippocampal/parahippocampal cortex; heightened DC values in the left PreCG and left SMG; and significantly reduced FC strength between the left PCC and regions including the left anterior cingulate cortex and left hippocampus. These abnormalities are closely associated with attentional deficits, aberrant self-referential processing, and impairments in episodic memory recall.

Concurrently, functional impairments within the VN contribute to diminished visuospatial construction, reduced figure retention, and compromised primary integration of visual information in COPD patients. Key manifestations include decreased ReHo values in the left occipital lobe and right lingual gyrus, reduced DC values in the right lingual gyrus, and weakened FC synchronization between the right lingual gyrus and both the left lingual gyrus and left fusiform gyrus. Additionally, dynamic cooperative efficiency between the VN and SN is significantly reduced. Notably, dysfunction extends to other critical networks: the frontoparietal network (FPN), which supports executive control and working memory, and the SN, responsible for attentional allocation and task-switching. These networks collectively exert significant effects on observed deficits in planning, abstract thinking, and cognitive flexibility. Critically, the global cognitive decline in COPD patients arises not only from intra-network abnormalities but is also intrinsically linked to dysfunctional coordination between networks (e.g., DMN-FPN, DMN-SN, and VN-FPN interactions). This disruption of inter-network coordination, potentially mediated by impaired global brain communication or compromised hub node integrity, reflects a systemic, network-level pathophysiological mechanism underlying COPD-CI. COPD-CI fundamentally stems from interconnected multidimensional pathological cascades.

Chronic hypoxia serves as a primary driver by directly inducing hippocampal neuronal pyroptosis, synaptic plasticity impairment, and BBB disruption. Neuroimaging evidence primarily found that reduced neural activity in bilateral basal ganglia and thalamus exhibits positive correlation with PaO₂. The severity of hypoxia predicts progression risk of dementia. Systemic inflammation invades the CNS through three complementary mechanisms: direct transmembrane passage or receptor-mediated endocytosis and their signalling to trigger neuronal apoptosis; indirect microglial activation releasing ROS and inflammatory cytokines that extensively damage white matter microstructure; and the distinctive vagus nerve pathway transmitting lung-derived inflammatory signals to higher cognitive centers like the ACC and PFC, establishing neuro-immune circuits. Notably, there seem to be some region-specific vulnerabilities: elevated TNF-α/IL-1β in the hippocampus disrupts episodic memory consolidation, cortical TNF-α overexpression interferes with executive integration, while the amygdala displays localized inflammatory dysregulation with microglial abnormalities.

Comorbid conditions markedly amplify cognitive damage. Specifically, OSA reduces MMSE scores via persistent hypoxemia, while cardio-metabolic disorders induce cerebrovascular microenvironment disruption, and depression/anxiety disorders exacerbate visuospatial and executive dysfunction. Smoking exposure demonstrates paradoxical effects: nicotine’s potential protection of dopaminergic neurons may partially improve cognitive metrics, though electronic cigarettes unequivocally impair memory retention. On the basis of these findings, long-term oxygen therapy, exercise training, and anti-inflammatory interventions may serve as potential strategies to improve cognitive function.

However, current research on brain network alterations in COPD-CI faces several key limitations, which can be examined from methodological, technical, and theoretical perspectives. Methodologically, most existing studies are based on cross-sectional or correlational designs. While these have identified associations between altered brain network metrics and cognitive decline, they do not establish causality. Furthermore, the focus has largely been on static functional connectivity, with limited exploration of dynamic temporal changes in network organization. Technically, fMRI presents specific constraints. The BOLD signal, as an indirect measure of neural activity, is susceptible to non-neural influences commonly seen in COPD patients—such as vascular dysfunction, cardiopulmonary physiology, and medication use—which complicates the interpretation of group differences. In addition, while fMRI is well-suited for mapping functional connectivity, it offers limited insight into the underlying structural or molecular pathology. Commonly used metrics such as FC, DC, ALFF, and ReHo reflect regional brain activity or synchronization but lack directional information, thereby restricting inferences about neural signaling pathways. Theoretically, the absence of a unified understanding of COPD-CI mechanisms has led to a predominance of data-driven approaches, with relatively few hypothesis-driven studies to provide conceptual guidance or mechanistic insight.

## Data Availability

No datasets were generated or analysed during the current study.

## Abbreviations

CI: Cognitive impairment
COPD: Chronic obstructive pulmonary disease
COPD-CI: COPD-related cognitive impairment
fMRI: Functional magnetic resonance imaging
DMN: Default mode network
VN: Visual network
FC: Functional connectivity
AD: Alzheimer’s disease
MoCA: Montreal Cognitive Assessment
CIH: Chronic intermittent hypoxia
BBB: Blood-brain barrier
PaO₂: Arterial oxygen partial pressure
ALFF: Amplitude of low-frequency fluctuation
dALFF: Dynamic amplitude of low-frequency fluctuation
CNS: Central nervous system
NF-κB: Nuclear factor kappa-light-chain-enhancer of activated B cells
IL-1β: Interleukin-1β
TNF-α: Tumour necrosis factor-α
IL-8: Interleukin-8
ROS: Reactive oxygen species
IL-6: Interleukin-6
ReHo: Regional homogeneity
OSAS: Obstructive sleep apnoea syndrome
MMSE: Mini-Mental State Examination
ECN: Executive control network
SN: Salience network
mPFC: Medial prefrontal cortex
ACC: Anterior cingulate cortex
CSE: Cigarette smoke exposure
PCC: Posterior cingulate cortex
L-PCC: left posterior cingulate cortex
PreCG: Precentral gyrus
SMG: Supramarginal gyrus
DC: Degree centrality
R-PHP: Right parahippocampus
SMA: Supplementary motor area
R-PCL: Right paracentral lobule
SMN: Sensorimotor network
LN: Language network
AN: Auditory network
dlPFC: Dorsolateral prefrontal cortex
dFNC: Dynamic functional network connectivity
LTOT: Long-term oxygen therapy
CAT: COPD Assessment Test
cAMP/PKA/CREB: Cyclic adenosine monophosphate/protein kinase A/cAMP response element-binding protein
BDNF: Brain-derived neurotrophic factor
FPN: Frontoparietal network
rs-fMRI: Resting-state functional magnetic resonance imaging
